# Practice of sustainable fashion design considering customer emotions and personal tastes

**DOI:** 10.3389/fpsyg.2022.976471

**Published:** 2022-10-04

**Authors:** Seonju Kam, Youngsun Yoo

**Affiliations:** Department of Clothing and Textiles, Kyung Hee University, Seoul, South Korea

**Keywords:** sustainable fashion, sustainable design approach, sustainability, SFB, emotional durability

## Abstract

This study aimed to determine a sustainable design practice approach that can satisfy customer emotions and personal tastes, which designers need in the early stages of the SFD process, and improve environmental performance. The research was conducted through a case study and interviews. For case studies, the specific design methods of fashion brands, which have been ranked sustainable over the last 3 years in the world’s top fashion magazines favored by the public, were researched. The results of the case studies were used to draw questions for the in-depth interviews. The results are as follows: first, the design approaches of SFBs were categorized into “eco-friendly materials,” “functional durability design,” “reuse and remanufacturing,” “emotional durability design,” and sustainable fashion technology. Each type’s specific design approach methods were organized into a checklist for the practice of SFD and then reflected in the interview questions. From the results of the interviews, it was noted that the sustainable design approaches perceived by Korean designers were “eco-friendly materials,” “reuse and remanufacturing,” and “functional durability design.” Moreover, it was mentioned that specific methods of emotional durability design and sustainable fashion technology need to be acquired. By applying the checklist to the interviewees, interview participants could conveniently and quickly recognize how to apply sustainable design through the inventory. This study is significant because it presents a checklist, an efficient tool for sustainable design approaches, and a sustainable design practice method that can satisfy customer emotions and personal tastes and improve environmental performance.

## Introduction

The fashion industry is one of the industries that have contributed significantly to the growth of the global consumer goods industry for decades. Nevertheless, the environmental damage caused by water pollution and CO2 produced at each stage of the fashion supply chain is the second largest after the oil industry (Villemain, 2019). Hence, the fashion industry’s responsibility for sustainable environmental development and its obligation to restore the environment are emphasized, as much as the share of the fashion industry in the global industry (Caniato et al., 2012; Dissanayake and Sinha, 2015; Lawless and Medvedev, 2016; Maldini et al., 2019; O’Connell, 2020). Since the mid-2000s, industrial supply systems around the world have been affected by sustainability and have struggled to develop environmental management strategies (Reoberto and Esposito, 2016). Previous studies have stated that a green supply system based on a circular economy is important in presenting a vision for sustainable manufacturing (Zhu et al., 2011; Stahel, 2016; Geissdoerfer et al., 2017). H&M has regularly published public reports on sustainability activities since it launched an ethical fashion brand called “Conscious Collection” in 2011 (Baker, 2011). In addition to mainstream brands such as Nike and M&S, it is considered a leader in sustainable business execution (Kozlowski et al., 2019; Claxton and Kent, 2020). Many fashion companies, including Uniqlo, North Face, and New Balance, also recognize the importance of sustainability and supply chain management (Shen, 2014). Early studies on sustainable fashion focused on eco-designs, which focused on the environmental harm during the product life cycle, from using materials to production and disposal. They were followed by studies on various tools for measuring performance in the three aspects of sustainability and strategies for sustainable fashion design (SFD) (Pigosso et al., 2013; Rossi et al., 2016; Ahmad et al., 2018; Karell and Niinimäki, 2020). Emotionally durable design aims at a circular economy as a design approach that extends the life of a product by encouraging a more durable and resilient relationship with the product through the emotional experience that occurs between the product and the consumer (Haines-Gadd et al., 2018). It can be said that it is a design method that allows modern people who consume selectively and wisely to choose sustainable product design according to their sensibility and personal taste. In previous studies, consumers agreed to the practice of sustainability but rejected sustainable products that did not fit their tastes (Karell and Niinimäki, 2020). Additionally, while about 80% of sustainability impacts are determined at the design stage, which is an early stage in the production process, design methods still tend to rely on the designer’s intuition (Ramani et al., 2010; Ribeiro et al., 2013; Ahmad et al., 2018; Keshavarz-Ghorabaee et al., 2019; Karell and Niinimäki, 2020). Designers play an essential role in sustainable environmental performance and decisively impact the future environmental effects of their products (Boks, 2006; Ramani et al., 2010; Ribeiro et al., 2013; Ahmad et al., 2018; Keshavarz-Ghorabaee et al., 2019; Karell and Niinimäki, 2020). Nevertheless, fashion designers still need to understand the complexity of sustainable fashion issues and the unpredictable future of fashion design related to diversity, rapidly changing trends, and consumers (Kozlowski et al., 2019). The world’s well-known fashion magazines, such as Vogue, Elle, and Harper’s Bazaar, rank and release articles on fashion products of sustainable fashion brands (SFBs). This implies that the public interest in sustainable fashion products is high. Thus, it is imperative to propose practical methods for easy-to-use SFD, in which the complexity of sustainability and the intuition and experience of designers are objectified.

The purpose of this study is to support the circular economy by satisfying customers’ sensibility and personal taste, improving environmental performance, and determining a design approach that designers can easily use in SFD.

## Literature review

### Sustainable fashion design

Sustainability means that businesses must address social goals such as environmental conservation, social justice, and economic development (Yıldızbaşı et al., 2021). It is in the same vein as the importance of business performance measured by considering the three dimensions of sustainability in the overall green industry (Pattnaik et al., 2021). SFD refers to design that considers the social, environmental, and economic impacts associated with the fashion products in the entire life cycle until the end of their life, from the raw materials to the use and disposal (Niinimäki, 2006; Kozlowski et al., 2019). Ecological, economic, and social factors have been the basis of many studies as the triple bottom line (TBL) of sustainability (Raza et al., 2021). Today’s SFD has evolved into a system that plans products to suppress the occurrence of environmentally hazardous elements in the fashion product supply chain (Ceschin and Gaziulusoy, 2016; Kozlowski et al., 2019). In the fashion industry, three out of five apparel items are discarded within a year of production (Puspita and Chae, 2021). Problem-solving in sustainable fashion requires improving the complex apparel supply chain and the consumers, companies, and governments involved. Several previous studies have noted that designers are crucial to influencing changes in the sustainable design industry (Lawless and Medvedev, 2016; Hur and Cassidy, 2019; Kozlowski et al., 2019). To achieve the sustainability goals of fashion products, designers should play an active role in design from the early stage of the production process by predicting the ethical behavior of fashion product production and consumption (Ceschin and Gaziulusoy, 2016). For SFD, Kozlowski et al. (2019) stated that aesthetic and cultural dimensions should also be considered along with performance in three aspects: the environmental, social, and economic aspects of sustainability. These aspects must be regarded because sustainable fashion products that have been produced so far have become another environmentally hazardous factor because they have not been chosen as consumers’ tastes are not met. Currently, various tools are used to predict the performance of sustainable fashion supply chains (Bovea and Pérez-Belis, 2012; Kozlowski et al., 2019). However, considering that approximately 80% of the sustainability impact over the entire life cycle of fashion products are determined in the design stage (Ribeiro et al., 2013; Ahmad et al., 2018), it is necessary to explore various approaches to SFD.

### Sustainable fashion brand

Fashion companies such as Zara, Nike, and H&M, including Kering, which currently has a portfolio of luxury brands, regularly publish public reports describing their sustainability activities (Kozlowski et al., 2019). Most sections of the fashion industry, such as general apparel, sportswear, shoes, and underwear, are paying attention to sustainable product development in consideration of environmental, economic, and social issues. In 2010, H&M announced the first sustainable collection made from sustainable materials such as organic cotton, linen, recycled polyester, and Tencel of wood pulp fabric (Portuguez, 2010). Then, in 2011, it launched a new “Conscious” collection and pledged to develop the Sustainable Apparel Coalition, an initiative devised to expand the use of organic and sustainable materials, educate cotton farmers, and measure the environment, impact, and labor practices for apparel and shoe manufacturing (Baker, 2011). In 2011, Patagonia also started the “Do Not Buy This Jacket” campaign, which promotes conscious buying, upcycling, and product use changes (Bandyopadhyay and Ray, 2020). Simultaneously, Patagonia operated a recycling program called the Common Threads Initiative, which focused on the “4 Rs” to enable the recycling of its products. It aims to reduce resale through eBay and recycling based on customer partnerships (Patagonia Inc, 2011). One of the interests of Patagonia was in ethics for the life of workers, and Patagonia became one of the first fashion brands to take responsibility in partnership with Fair Trade USA. This movement has advocated for improved social and environmental standards since 2014 (Teen Vogue, 2019; Bandyopadhyay and Ray, 2020). In 2014, to develop a roadmap to create a more sustainable supply chain and conserve endangered forests in Ho Chi Minh, Vietnam, Stella McCartney, H&M, Eileen Fisher, Patagonia, and Inditex/Zara formed a group of promising forest conservation policies. The group created a shared “knowledge map” for the viscose supply chain to facilitate the removal of endangered forest fibers and pledged to support a long-term conservation solution for high-priority forest areas, such as rainforests in Indonesia and rainforests and subarctic forests in Canada. Furthermore, they have pledged to support the development of sustainable fabric alternatives made of recycled fabrics, recycled materials, and agricultural byproducts such as straw (Sustainable brands, 2014). Stella McCartney is a London-based luxury brand belonging to Kering that does not use unsustainable animal materials, such as fur, leather, and feathers. It is known to operate a brand with ceaseless sustainable thinking. Their 2019 collection was rated as the most sustainable among the past collections because 75% of the collection used Econyl and recycled polyester, while the rest used organic cotton or upcycled denim. They announced Koba faux fur made from corn byproducts mixed with recycled polyester as an alternative to plastic options (Frost, 2019).

In 2015, Kering announced Environmental Profit and Loss (EP and L), a sustainability statement calling for industry accountability. In 2016, EP and L were applied to all brands of Kering. Further, the EP and L demanded environmental and ethical responsibility across the supply chain from damage to environmental impacts caused by fashion products and not to evade fair-trade labor practice, carbon imprint, and energy and resource conservation (Social Media Today, 2015). It started with upcycling fashion brands in 2008 and evolved as Kolon Industries, a large fashion company, launched “RE: CODE,” an upcycling fashion brand that introduced fashion products manufactured by recycling fashion products to be incinerated and automotive parts (Park and Kim, 2014). RE: CODE was launched in 2012 as a sustainable brand by Kolon Industries, Inc., a large fashion company in South Korea. It creates new value based on upcycling, which refers to making new clothes by recycling deadstock and clothing waste. RE: CODE breaks fashion stereotypes, creates new uses, and encourages the world to participate in environmental and sustainable societal movements (Kolon Industries, 2012). Kolon Industries has been working on the Noah Project since 2016 as a campaign to protect endangered animals and plants in South Korea. “Kolon Sports” of Kolon Industries applied 100% eco-friendly materials and techniques to all products in the collection in 2020 as part of the Noah Project (Park, 2020).

As described above, the sustainable activities of fashion companies are group activities and campaigns focused on eco-friendly materials and material recycling. More and more fashion brands were putting the concept of sustainability at the forefront of their design goals.

### Sustainable design approach and method

Previous studies have dealt with guides for various conceptual design tools and strategies to help apparel designers implement sustainability. Ceschin and Gaziulusoy (2016) classified sustainable design approaches and methods into “green design and eco-design,” “emotionally durable design,” “nature-inspired design,” “cradle-to-cradle design,” “biomimicry design,” “design for the base of the pyramid,” “sustainable product-service system design,” and “design for system innovations and transitions.” Rossi et al. (2016) summarized design approaches with “design for X concept” and classified them into “design for disassembly,” “design for remanufacturing,” design for material recycling, and “design for energy efficiency.” Based on some previous studies, Irwin (2015) and Sumter et al. (2020) classified design approaches by adding “design for a circular economy” to “eco-design,” “nature-inspired design,” “sustainable product-service systems,” “design for low resource settings,” “design for social innovation,” and “transition design.” De Pauw et al. (2014) conducted exploratory case studies to compare “eco-design” as an eco-friendly method to the methods of “biomimicry” and “cradle-to-cradle.” Väänänen and Pöllänen (2020) stated that the introduction of craft techniques into recycling and upcycling products makes products aesthetically pleasing and meaningful, which can be associated with the emotional durability of products that increases consumer attachment. Attachment can be one of the solutions to these problems because sustainability products in the past have not elicited empathy for respecting the individualities and tastes of consumers, compared to the increase in environmental awareness among consumers (Karell and Niinimäki, 2020). Ramani et al. (2010) have classified “modular design,” “part standardization,” “take-back management,” “design for disassembly,” “design for reuse and remanufacturing,” and “design for material recovery” as design methods for improving end-of-life (EOL) management that enables multiple life cycles of “cradle-to-cradle.” Ramani et al. (2010) mentioned developing a laser-based manufacturing process to reduce material waste. Further, it involves not releasing hazardous elements during design and processes using computer-aided design (CAD) and computer-aided process planning (CAPP), which can affect the design in the early stage.

**FIGURE 1:**
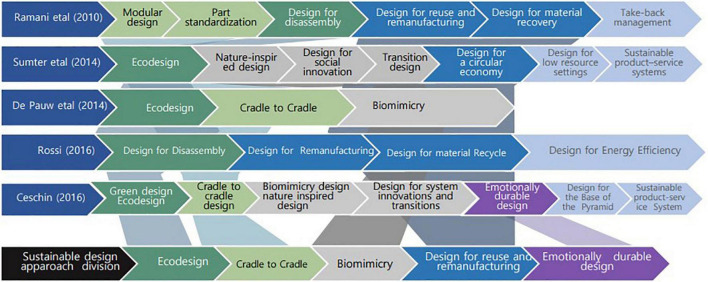
Process of classifying sustainable design approaches based on previous studies.

Figure 1 summarizes the classification of design approaches by researchers in previous studies. Based on these earlier studies on sustainable design, we classified design approaches into five categories in the early stage of sustainable design in this study. These include “eco-design,” “cradle-to-cradle,” “biomimicry,” “design for reuse and remanufacturing,” and “emotionally durable design,” which were used in the case analysis of sustainable designs in the next section. Figure 1 shows the process of deriving five sustainable design approaches based on the classifications of the five previous studies.

## Methodology

**FIGURE 2:**
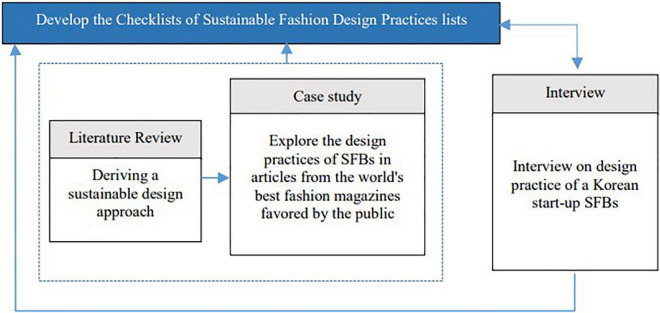
Framework of this study.

The research was conducted through a case study and interviews. The research procedure is (1) classifying sustainable design approaches through a review of previous research; (2) based on this, the sustainable design approach and detailed design method for fashion designers were investigated in the world’s top fashion magazines favored by the public, (3) using the results of the case study as a tool for an in-depth interview with designers of SFB in Korea, and (4) determining design approaches that designers can easily use in the early stages of the SFD process. Figure 2 illustrates the framework of the study.

### Case study

Regarding the research method, it analyzed the cases for the representation methods of SFBs that were ranked in the world’s top fashion magazines based on the sustainable design approaches derived through the literature review. The analysis focused on a total of 141 SFBs in nine articles searched using “the best SFB” in Vogue, Elle, and Harper’s Bazaar, which are the world’s top popular fashion magazines for 3 years from 2019 to 2021. Additionally, for the analysis of the design approaches of the collected 149 SFBs, additional design methods were identified in the introduction window and product introduction of brand websites, along with the contents of the articles. Table 1 summarizes the titles of the nine articles for the top-ranking fashion brands in the analyzed fashion magazines.

**TABLE 1:** Articles on sustainable fashion brands (SFBs) selected from the world’s top popular fashion magazines.

Name	Year	Title of the article
Vogue	2021	11 Brands Taking Positive Steps This Earth Day
	2020	25 Sustainably-Made Goods to Recharge Your Winter Wardrobe
	2019	29 Stylish and Sustainable Pieces to Reboot Your Fall Wardrobe
Elle	2021	55 Sustainable Clothing Brands That Are Anything But Boring
	2020	24 Sustainable Clothing Brands That Are Anything But Boring
	2019	22 Eco-Friendly Brands to Shop on Earth Day
Harper’s Bazaar	2021	Our favorite sustainable brands
	2020	Earth Day 2020: How Sustainable Luxury Brands Are Making a Change
	2019	How Sustainable Are These “Eco-Friendly” Fashion Brands?

### Interview

The interviews were conducted from 14 September 2021 to 30 March 2022. The interview participants were randomly selected from among the brands selected or applied for the SFB support project of the Korean or local government. Eleven designers from sustainable fashion start-ups in Korea participated in the interviews. Each interview was conducted face-to-face or *via* Zoom and lasted approximately 40–50 min. Table 2 shows the contents related to the interview participants, including Sustainable Fashion Branding Experience, Fashion Designer Experience, and fashion products designed by them. Letters were assigned according to the order of the interviews to ensure anonymity.

**TABLE 2:** Interview participants.

Interview participants	Sustainable fashion branding experience (years)	Fashion designer experience (years)	Products
A	4	10	Knit, jersey wear, bag
B	6	6	Women’s wear, men’s wear, accessory
C	3	3	Accessory, bag, women’s wear
D	4	22	Women’s wear
E	4	5	Women’s wear, men’s wear, accessory, shoes
F	2	2	Women’s wear, men’s wear, cap
G	2	2	Women’s wear
H	3	5	Bag, accessory, daily supplies
I	2	2	Pouch, accessory
J	2	5	Secondhand product reform, women’s wear, digital cloth pattern
K	12	15	Women’s wear

**FIGURE 3:**
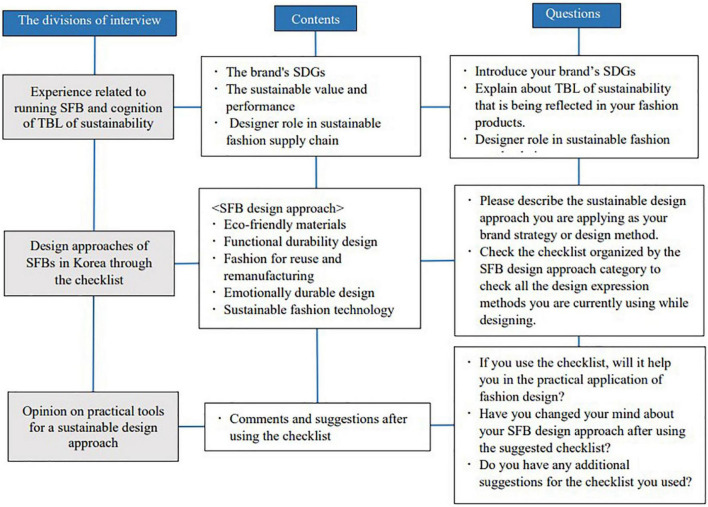
Interview questions on sustainable fashion brands’ (SFBs) design approach methods.

The interviews were recorded and transcribed with the consent of the interviewees. Semi-structured questions were used for the interview, and additional questions were asked to obtain specific answers and opinions. As shown in Figure 3, the interview questions were mainly composed of three questions. The first part concerned the launch date of SFB, the goal of sustainable development, and cognition of triple bottom line (TBL) of sustainability. The second part was to identify the difference between the design approach currently used by the interviewed designers and the design method shown in the world’s best fashion magazines favored by the public, through the SFB design approach checklist based on the case study results. Finally, the third part consisted of comments and suggestions on practical tools for a sustainable design approach after the interview participants had used the checklist. Figure 3 is the frame of the interview question extraction process based on the checklist derived from the case study.

## Results

### Case study of sustainable fashion brands’ design approach

**FIGURE 4:**
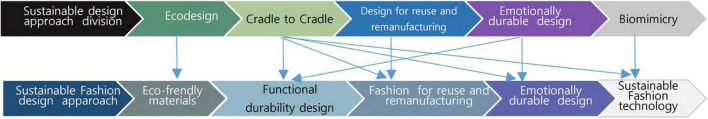
Design approach classification process of sustainable fashion brand (SFB).

A total of 149 SFBs were ranked by the world’s most popular fashion magazines for 3 years. Among them, 34 SFBs appeared twice or more, indicating that the SFB market has not yet been established stably. This may be an obvious result because it has only been approximately 10 years since fully fledged SFBs emerged. However, 35 brands were ranked only once in 2019, 19 in 2020, and 56 in 2021. Fashion brand activities were reduced in 2020 because of the SFB market shrinkage caused by COVID-19. Nevertheless, it can be seen that public interest in SFBs has increased since the number of new fashion brands in popular fashion magazines grew significantly in 2021. Thus, it is necessary to suggest a practical design approach for SFD that consumers can directly choose. Figure 4 shows the design classification process of the SFB based on the sustainable design approach classification derived from the literature review and was used as the category for the following case study.

As a result of the case analysis based on the sustainable design approach of the previous studies, the design approaches of SFBs were categorized into: “eco-friendly materials,” “functional durability design,” “fashion for reuse and remanufacturing,” “emotionally durable design,” and “sustainable fashion technology.” Furthermore, case analysis was conducted for the specific design approaches applied in the early stage of the design process of SFB based on these categories as follows:

#### Eco-friendly materials

The use of eco-friendly materials is one of the metrics of sustainable fashion. Specifically, as eco-friendly materials are used, the sustainability of each product increases (Wang and Shen, 2017). The environmental impact during the product life cycle can be minimized only by choosing eco-friendly materials (Ribeiro et al., 2013; Ahmad et al., 2018; Claxton and Kent, 2020). In particular, sustainable fashion products made of organic fabrics are fundamental to the supply chain because they contain fewer chemicals that harm the environment (Shen, 2014). At the initial design stage, designers should consider using biodegradable materials that can be returned to the soil without causing additional damage to nature (Gurova and Morozova, 2018).

The study of SFB product cases revealed that the selection of eco-friendly materials was required in almost all companies as a design approach. It appeared with eco-friendly materials, 100% organic cotton materials, a method tracing the origin of materials, or using vegetable materials. Additionally, it adopted a short-distance distribution to use eco-friendly materials near the production site as SFB’s design strategy to reduce CO2 emissions.

1. **Certified sustainable materials using 100% organic cotton materials** include Patagonia (Nagurney and Yu, 2012), H&M Conscious (Bédat, 2019), Stella McCartney (McCartney, 2020), Mara Hoffman (Bédat, 2019), and Theory (Elle Fashion Team, 2020), Burberry (Wang, 2020), House of Sunny (Davis, 2021), BITE Studios (Elle Fashion Team, 2021), Reformation (Bédat, 2019), Baserange (Elle Fashion Team, 2021), and Yasmina Q (Davis, 2021), among others.
2. **Tracing the origin of eco-friendly materials:** Stella McCartney has adopted a method of tracing the origin of trees supplying viscose raw materials used strategically to help the environment by protecting endangered forests (Davis, 2021) and, further, including those facilitating tracing of all eco-friendly materials on the brand’s website (Elle Fashion Team, 2021).
3. **Using vegetable materials:** Vegan materials include Bleusalt’s signature fabric, an entirely vegan material with beech (Penrose and Hearst, 2019). Moreover, notably, Alohas made shoes with two vegan types of leather from cactus and corn (Elle Fashion Team, 2021). VEJA’s sneakers used organic cotton for fair trade and soles made of rubber grown in the Amazon rainforest (Elle Fashion Team, 2021). Additionally, Allbirds often makes soles with sugarcane and manufacture uppers using eucalyptus or natural merino wool (Davis, 2021).
4. **Net zero:** Mulberry produces bags by developing the lowest carbon leather (Vogue, 2021). Sonia Carrasco uses only organic or vegan materials for clothes and tags, labels, packaging, and papers (Elle Fashion Team, 2021). Wright Le Chapelain maintained a transparent supply chain of sustainability and fabrics sourced from UK factories over short distances (Elle Fashion Team, 2021). Tretorn also launched eco-friendly sneakers made of locally sourced canvases (Davis, 2021).

#### Functional durability design

The properties and quantity of materials and the shape of the clothes used by fashion designers affect the quality and durability, which can remarkably impact the life of clothes (Claxton and Kent, 2020). Connor-Crabb et al. (2016) argued that *trans*-seasonal, multi-functionality, modularity, alterability, and physical emotional durability are approaches to functional durability design. Further, they stated that on-demand production is included in this category. According to Rahman and Gong (2016), functional durability design extends the physical life of durable, organic, and recyclable fabric materials from a technical perspective. Moreover, it is a method of extending aesthetic life based on the emotional durability of the product. This study separated the approaches to emotional durability and discussed them. Transformable apparel provides two or more functional or aesthetic alternative styles (Rahman and Gong, 2016) and can extend the life of clothes. Modularized garment design is the task of dividing a garment into several parts based on the functional analysis of different parts. As many examples of various functions and specifications are included in each piece, user-oriented clothes can be designed quickly and flexibly (Zhou et al., 2016). According to the case study, the method of functional durability design appeared to be on-demand production, quality, durability, multi-functionality, and alterability.

1. On-demand production: The House of Sunny works on only two seasonal collections per year and produces small quantities based on orders. The design team spends more time researching sustainable fabrics, manufacturing methods, and sourcing materials (Elle Fashion Team, 2020, 2021; Davis, 2021). Further, Maison Cléo minimizes waste by selling it only once a week (Elle Fashion Team, 2020). Mary produces timeless limited editions based on orders without inventory (Elle Fashion Team, 2021).
2. Quality and durability: Everlane has chosen the finest materials and manufacturing methods for timeless products, such as the highest class cashmere sweaters, Italian shoes, and Peruvian Pima t-shirts (EVERLANE, 2021).
3. Alterability: Misha Nonoo’s “Easy 8” collection features eight pieces that can produce 22 changeable looks (Davis, 2021). Nynne has included various styling options and is placed in a seam line across the leather skirt so that the length can be reduced if the user gets bored of the size and introduced reversible shearling jackets for two completely different looks (Davis, 2021). The CAES has proposed timeless items that can be worn throughout the year by adding a premium to slow fashion with a concept that compares clothes to protective “cases” that cover our bodies (Elle Fashion Team, 2021). Petit Pli designed clothes that can be worn for a long time, even if the body changes, by creating variable garments that can be increased or decreased in length depending on the wearer in a chic-pleated manner. Cho proposed varying designs with clothes that could be adjusted in size based on a detachable panel in the style of clothes manufactured using recycled plastic bottles and ethically sourced (Elle Fashion Team, 2021).

#### Fashion for reuse and remanufacturing

Energy is required for designing and producing new products (DeLong et al., 2014). Therefore, sustainable fashion designers should consider valuable new product design methods that facilitate multiple life cycles by reusing and reconstructing discarded products. Janigo and Wu (2015) classified design approaches for reuse and remanufacturing into repair and alteration, upcycle, downcycle, post-consumer used and secondhand clothing, post-consumer recycled clothing, and redesigned clothing. Gurova and Morozova (2018) stated that upcycling, reuse, and repurposing methods exist.

In the case study of the SFB approach, the methods of reuse and remanufacturing were sourcing sustainable yarns from waste, redesigning clothing, and repurposing.

1. Recycled yarns: Burberry heritage trench coats and lightweight classic car coats are produced using Econyl, a sustainable nylon yarn made of recycled fishing nets, fabric scraps, and industrial plastics (Wang, 2020). Baum und Pferdgarten uses recycled denim and recycled polyester from plastic bottles (Elle Fashion Team, 2021; Vogue, 2021). Maggie Marilyn sourced 100% of synthetic fibers discarded after consumption (Marius, 2020). Prada launched Prada Re-Nylon, a line of sustainable bags and accessories made of discarded cloth and recycled plastics collected from the sea and fishing nets (Elle Fashion Team, 2020). JW Anderson introduced belt totes made of recycled plastic (Elle Fashion Team, 2021). PAPER London launched swimsuits produced using recycled yarns from fishing nets, which would have taken 600 years to discompose (Elle Fashion Team, 2021). The Pringle of Scotland, known as knitwear, has used 100% recycled fibers to produce limited-edition jumpers and recycled clothing tags (Elle Fashion Team, 2021).
2. Redesigned clothing: Acne Studios has designed super-sized jackets and unique mini-skirts of modern images that the brand has as products that recycled discarded black denim and red leather (Elle Fashion Team, 2021). Rave Review introduced luxurious upcycled fashions using fabrics and deadstock clothes and created tufty overcoats by upcycling vintage bedspreads (Wang, 2020). Marine Serre has sourced discarded scarves, secondhand shirts, and wetsuit materials, turning them into futuristic practical wear from parkas to panel dresses (Lim, 2019).
3. Repurposing: Mulberry bags aim to extend product life through repair, restoration, buyback, reselling, and repurposing (Vogue, 2021). Matty Bovan sourced the fabrics and prints used in its collection by working with the Liberty Fabric Archives. In a previous collection, they recycled soccer pads to inflate the shoulders and redesigned old fur into new shapes (Bonacic, 2020).

#### Emotionally durable design

An emotionally durable fashion design approach can extend the product life cycle based on the emotional attachment between consumers and products (Claxton and Kent, 2020). Emotionally durable fashion originates from a business environment in which products connect consumers and manufacturers and provide conversation pieces that facilitate the ease of upgrades, services, and repairs (Chapman, 2005). Consumers are attached to physical objects through complex interactions between cultural norms, personal preferences, and behaviors (Connor-Crabb et al., 2016). Fashion customers with a taste for handcrafted and luxurious products are emotionally attracted to secondhand clothes reborn with felt, quilt, and dye and purchase them (Janigo and Wu, 2015). Consumers stay attached for longer to products that elicit amazement and endless pleasure (Armstrong et al., 2016). Consumers’ attachment to products that meet their personal characteristics and tastes leads to an extension of their product life. Design strategies that encourage social contact through sharing or group use may lead to attachment (Armstrong et al., 2016). Upcycling designs using heirlooms or garments with strong personal attachment have emotional durability (DeLong et al., 2014). Furthermore, handicrafts made by artisans have substantial value as a medium of sustainable fashion with devotion, as sustainable design reflecting local resources and culture can lead to the derivation of narratives (Sandhu, 2020).

In the study of SFBs, emotionally durable fashion designs appeared to collaborate with artisans and artists in the production area, handwoven material sourcing, and emotional design concepts.

1. Collaboration with artisans: Bite Studios creates sustainable fashion products by collaborating with emerging and existing artists in various works, such as natural dyeing techniques, printmaking, and handmade jewelry (Vogue, 2021). Chopova Lowena (Elle Fashion Team, 2021) pursues uniqueness with vibrant combinations of Bulgarian folk handcraft materials made through craftsmanship and English tailoring (Elle Fashion Team, 2020; BROWNS FASHION, 2021). Hereu’s bags and shoes are products made by local artisans at the home of the founding designer of Spanish nationality (Elle Fashion Team, 2021). Ballen Pellettiere accessories commemorate Colombian fashion and artisans’ crafts, and playful embroidery paired with a unique shape is a trademark of their handmade bags (Penrose and Hearst, 2019).
2. Handwoven material sourcing: Bethany Williams’ recycled tents and handwoven denim ensembles reflect their signature multicolor patchwork and streetwear sentiments (Lim, 2019), while wooden buttons handcrafted by carving are discarded birches that reflect consumers’ individualities and preferences (Bonačić, 2021). Bodes are brands that use recycled vintage cloth as materials and have unique handcrafted works containing stories of quilting, mending, and appliances by sourcing fabrics from all over the world, including Victorian quilts and 100-year-old linens (BODE, 2021). Brother Vellies’ shoes and handbags are handmade in South Africa, Ethiopia, Kenya, and Morocco, combining the expertise of local artisans.
3. Personal design concept: Nynne approaches sustainable fashion consumer sentiment with a unique design concept named “Diana” dress as the brand’s signature work (Davis, 2021).

#### Sustainable fashion technology

Digital tools can be used to find new behaviors in existing materials by modifying their structures, and a new understanding based on this can expand the possibilities provided to designers. By extensively using 3D design software, designers can design complex woven clothing, even if they have little understanding of weaving or weaving software (Chapman, 2005). Sustainable fashion technology is related to creative pattern cutting, which can reduce environmental impact. Zero-waste pattern cutting is making fabric using the predetermined width and length to minimize the fabric’s loss in the cutting stage (Townsend and Mills, 2013). Zero-waste fashion can show new expressions while reducing or eliminating waste in product production by mixing creative design practices and zero-waste pattern cutting (McQuillan, 2019). Applying this method requires intuition and experience. However, in recent years, innovative designs and technological progress have made it easier to adopt creative practices. Software such as CLO enables fast initial design creation and facilitates the development of highly innovative woven shapes by visualizing 2D patterns, 3D shapes, and waste generated during garment design (McQuillan, 2019).

In this study, the zero-waste fashion approach also included cases in which technologies that did not affect a sustainable environment were utilized.

1. 3D technique: PRISM Squared swimwear, sportswear, underwear, and shapewear produced by a seamless 3D knitting technique are created with almost no loss of fabrics during the production process (Elle team, 2020).
2. Digital printing: Hoffman performs digital printing directly on finished sweaters to ensure that the loss of fabric caused by pattern matching will not occur (Marius, 2020; Offman, 2021).
3. Lasers and robotics: Levis produced jeans in a way that is better for the environment by combining lasers and robotics (Elle Fashion Team, 2020; Davis, 2021).

#### Checklist from the result of the case study

**FIGURE 5:**
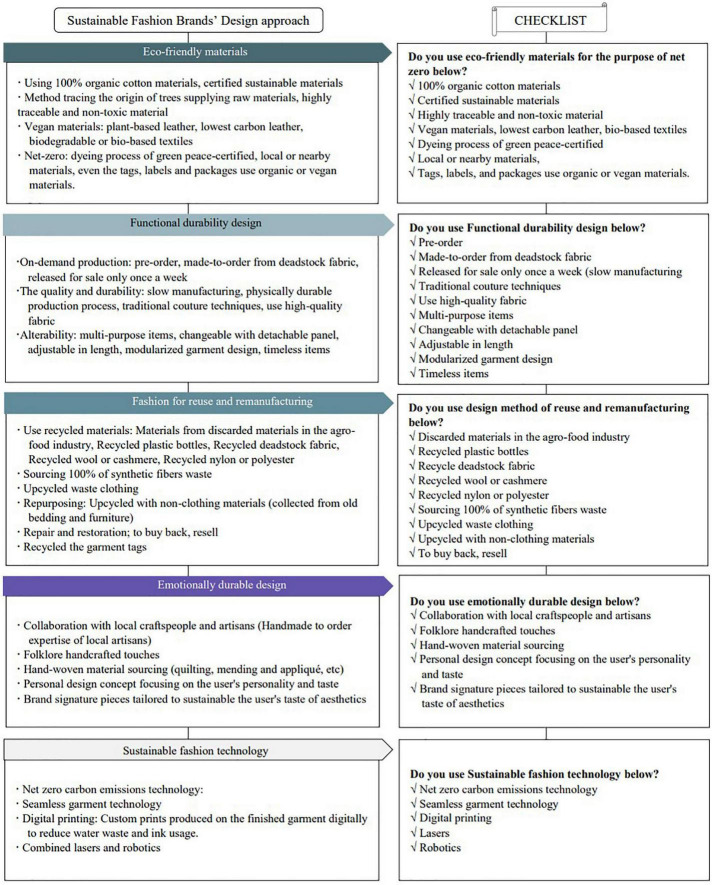
Representation methods by sustainable fashion brand (SFB) design approach category.

Figure 5 shows a summary of the specific methods for each design approach category, which can be applied in practical design in the early design phase of SFBs based on the experimental techniques derived from the case studies for each SFD approach category.

### Interview

The interview was conducted in three stages. In the first stage, questions were about fashion designers and SFB practical experience, cognitions related to TBL of sustainability, and whether and how TBL performance was applied to the company. In the second stage, an interview was conducted to find out the current practical approach of the interviewees using the SFD approach checklist derived from the SFBs case study results that appeared in the world’s top popular fashion magazines. The third stage was an interview on whether the checklist can be used as a practical tool for a sustainable design approach. Eleven brands participated in interviews.

#### Experience related to running a sustainable fashion brand and triple bottom line of sustainability

Designers can have a significant impact on the environment by intervening early in the sustainable fashion industry supply chain. With this in mind, the first question was about knowledge of TBL and designer experience. The brands participating in the interviews ranged from micro-sized companies with one person to small- and medium-sized companies with fewer than ten employees. The duration of the SFB operation of the interviewees was between 2 and 12 years. Some of the interviewers were aware of the value and performance of the TBL of sustainability and able to properly explain the application cases in practice. The others could explain corporate SDGs, but misunderstood the TBL of sustainability. That is, most interviewees were aware of environmental values, whereas some had difficulty approaching economic and social values. In particular, they misunderstood the economic value of sustainable environmental development as the economic performance of the company. This is consistent with previous studies in which designers discussed inadequate knowledge about sustainability and the lack of time to acquire it (Knight and Jenkins, 2009; Bovea and Pérez-Belis, 2012). The results support that tools for a sustainable design approach should be designed as effective learning mechanisms.

“From the social aspect of TBL, we actively hire women who have lost their careers to provide jobs for women who can be marginalized. From an environmental point of view, the use of recycled plastic bottles was actively introduced in all of the brand’s products, design, manufacturing method, and packing. We strive to reduce the impact of the environment through disposal, end-of-life treatment, which also contributes to sustainable environmental development and economic performance.” (Interviewee A)

This interviewee’s case was characteristic in that it aimed to expand the use of recycled plastic bottles. On the other hand, Interviewee D argued “to minimize the environmental impact, even plastic should not be used.”

Interviewee A and D had opposite views of sustainable development. In the report “Synthetics Anonymous” released by the Changing Markets Foundation (2021), it is noted that downcycling plastic made from recycled plastic bottles, that is, clothing using recycled polyester, will eventually end up in landfill or incineration rather than circulating fashion. The use of PET bottles as a material for recycling is expected to be controversial in the future.

The role of designers is to create an opportunity to increase the sustainability of fashion design. Further, it is a critical change agent in sustainable fashion (Niinimäki and Hassi, 2011). Most interviewees were aware of the importance of the designer’s role in attaining the value of sustainability. Interviewees A, B, C, D, and E discussed the importance of designers in reaching the value of sustainability because designers influence the life cycle of fashion products, and the design process is organically intertwined with all other areas. Interviewee I explained that a designer’s sense of design determined customers’ product selection and utilization. Moreover, they discussed the importance of design considering customer emotions and personal tastes to induce consumption of sustainable fashion products. Interviewees F and K stated that the role of designers is to convey the importance of sustainability to customers or boost sustainability in customer emotions and personal tastes. Through the interview results, designers can reflect on customer emotions and personal preferences in sustainable fashion products and exert influence throughout the design process to achieve sustainable goals. Designers can effectively implement sustainable fashion if there are tools that make the sustainable design approach more specific, practical, and easy to use.

#### Design approaches of sustainable fashion brands in Korea through the checklist

The interview on SFB’s approach to sustainable design practice in Korea was conducted by presenting a checklist derived from the case analysis results in the previous chapter. As a result of participating in the checklist, the SFD approach of the brands which participated in the interview mainly utilized “eco-friendly materials,” “functional durability design,” and “fashion for reuse and remanufacturing.” Some brands were new to or unfamiliar with the detailed expression methods of “emotionally durable design” and “sustainable fashion technology.” However, it is thought that it will be helpful for the expansion of sustainable design approaches in the future by realizing that the design process that is currently being implemented for customizing consumer tastes and the design inspired by their own culture belong to this area during the interview. The “eco-friendly materials” design approach is the design approach that most interviewees used, and there were various design expression methods. For example, Interviewee B used leather from the mulberry bark or cactus. Conversely, Interviewee D used sustainable materials, such as organic linen produced even on land unsuitable for grain production with low water consumption and pollution, and GOTS-certified organic cotton. Most of the brands interviewed chose green materials as a sustainable design approach, similar to a case study of SFB products presented by the world’s leading fashion magazines that are popular with the public. However, there was no mention of a method of tracing the origin of eco-friendly materials or tracking the use of eco-friendly materials at a short distance, which is a specific design approach shown in the results of the case study.

In the case study of fashion magazines, “functional durability design” presented specific design methods such as a pre-order method without stock, quality and design that can be worn over time, high-quality sewing, and a manual showing various styling with the few fashion items. Similarly, SFBs in Korea used manual finishing and preorder on-demand methods to ensure the robustness of their products and taught them various styling methods and easy repairs.

”As a company that produces sustainable bags and clothing, it enhances the solid finish with high-quality sewing using hand-sewn in the final finishing process.” (Interviewee B)

”We are adopting the slow business model as a seasonal, non-fashionable design method.” (Interviewee C)

“By connecting the small-volume production method of preorder with brand membership, we create a customer group with high loyalty to the brand. This avoids unnecessary production, resulting in environmental and economic performance. It gives advice to consumers on styling when they cannot use the purchased product and provides customers with information on laundry and care. Buying well-made products from good materials will extend the lifespan of your clothes.” (Interviewee K)

In the case study, “fashion for reuse and remanufacturing” was shown to be resourcing sustainable yarns from waste, or redesigning and repurposing. That is, recycled fishing nets, pieces of cloth, fabrics resourcing from plastic bottles, vintage clothing, outworn bedding, etc., were recycled and redesigned, and the original use of the material was changed. Similarly, in Korea’s SFB interviews, “fashion for reuse and remanufacturing” was found to use resourced materials from waste plastic bottles, use scrap or stock fabrics, or recycle discarded clothing. Among the design expression methods shown in the case study results, most expression methods were used by the brands participating in the interviews, except for recycling waste generated in the agro-food industry as a material.

“In Korea, the domestic waste plastic bottle market is active and has been developed using various materials. So, companies who want to use it can easily purchase it.” (Interviewee A)

“Among the clothes purchased from our brand, we collected the clothes the customer wanted to discard and upcycled it in the direction the customer wanted. The customer liked it very much.” (Interviewee K)

“We are producing hand-knitted handbags by collecting materials thrown away during the clothing-making process.” (Interviewee G)

“The main item is a fabric book cover, and the direction of our brand is to collect discarded scraps and waste subsidiary materials and recreate them as marketable products using handicraft techniques.” (Interviewee I)

“We produce and provide digital patterns that are used to remodel used clothing and provide tutorials for redesigning used clothes into clothing and accessories.” (Interviewer J)

In some cases, wastes with poor function for sports or leisure were recycled and developed into clothing.

”Leisure sports materials such as paragliding, glamping, tents, sails which have been destroyed for safety reasons, but have no problem in actual use, are collected, dismantled, washed, and recycled through a series of processes such as cutting and sewing.” (Interviewer H)

As mentioned above, the approaches of “emotionally durable design” and “sustainable fashion technology” were utilized in conjunction with “functional durability design” or “fashion for reuse and remanufacturing.”

“Emotionally durable design” was a method recognized and applied by only a small number of brands participating in the interview. Interviewee E understood that this design approach is sustainable after checking the design approach of “emotionally durable design” in the checklist.

The specific method of “emotionally durable design” shown in the case study was collaboration with local craftspeople and artisans, folklore handcrafted touches, handwoven material sourcing, personal design concept focusing on the user’s personality and taste, and brand signature pieces.

In Korea’s SFB interviews, “emotionally durable design” appeared as an inspirational approach to handicrafts such as knitting, quilting, and traditional elements of Korea.

“Through work that mixes handicraft with everyday products, we want to appeal to consumers’ sensibility and emphasize to consumers that everything from cutting to finishing is done manually.” (Interviewer I)

“Because we produce products using the preorder method of “Saekdong,” a traditional Korean element, as our brand signature item, we can reflect the individuality and taste of consumers.” (Interviewer E)

“Sustainable fashion technology” is the design approach adopted the least by the brands that participated in the interview. Although it was recognized as a sustainable design approach, designers faced barriers. This was consistent with a previous study, establishing that designers are limited in their adoption of tools for sustainable design or are unable to use them because they are unaware of their existence (Kozlowski et al., 2019). Among the brands that participated in the interview, Interviewees A and B, whose company size was large, actively used seamless 3D knitting techniques, digital printing, and laser cutting technology for finished fashion products but did not mention robotic technology.

“We know that digital printing technology is a sustainable fashion technology, but it is economically burdensome for our head office to have digital printers.” (Interviewee F)

“Our brand also produces knits and jerseys; thus, we know 3D knitting is a comfortable and sustainable way to wear it, but do not know how to approach it.” (Interviewee G)

“Sustainable fashion technology” had a high barrier for interview participants to approach. This is because the size of the brands participating in the interview was small. Notwithstanding, considering that the scale of SFBs is small- and medium-sized, sustainable fashion technology is a sustainable design approach that requires active support or investment from the government.

#### Opinion on practical tools for sustainable fashion design approach

After using the suggested checklist, interviewees were asked for their opinions and suggestions on the checklist as a practical tool. Regarding the advantages of using the inventory, the interviewees stated that the checklist, a valuable tool for a sustainable design approach, can help clarify a brand’s strategy and easily learn sustainable design approaches. This is considered a tool that can overcome the barriers and limitations of the sustainable design approach. Additionally, they stated that the direction of the sustainability concept could be identified more clearly if the checklist was used when establishing a sustainable brand strategy or planning a new product that pursues sustainability.

“The checklist provides guidelines for SFD. I thought it existed only in theory, but if I checked it when making a product, one could address the missing parts. Furthermore, sustainable brands pursue different goals. It is helpful to think about which side we focus on and value more.” (Interviewee C)

“It is an opportunity to check the brand design direction once more while checking the checklist.” (Interviewee F)

“I think I can check the brand concept by looking at this checklist when doing a new project.” (Interviewee H)

They said learning new sustainable design expression methods is also an advantage.

“While going over the checklist, I thought sustainability could be expressed this way. If we focus on what we are doing in practice, there would be insufficient time to review other things. Thus, the checklist can enable easy and quick understanding.” (Interviewee B)

“I was worried about not practicing it or overlooking it because of ignorance. If there is a tool that is easily accessible like this, I believe it would be convenient to practice.” (Interviewee G)

The advantage of the checklist mentioned by the interviewees is that it enables them to recognize the goals of sustainable development and clarify the design approach according to the concept of the brand. Moreover, the checklist is a tool for effectively learning the design approach to sustainability.

As suggestions for the checklist as a practical tool, constant updates, quantification for objective verification, and more in-depth details were mentioned.

“It seems that new ways to express design that pursue sustainability are emerging as time passes. New methods are proposed yearly for ease of recycling and economy, such as using single-component materials, design, and manufacturing that are easy to repair, reward policy, and lightweight to reduce carbon emissions. Therefore, new methods must be updated over time.” (Interviewee A)

“When it comes to dyeing, the abuse of water becomes a problem. I have encountered a dry dyeing technique that saves approximately 90% of water use, but it is not on the checklist. It would be good if new methods are constantly updated.” (Interviewee F)

Brand A participating in the interview presented numerical values for objective verification.

“Among famous overseas sustainable brands, there are brands that numerically represent sustainability. There is an objective feeling that numbers give. It shows the depth of our participation in sustainability together.”

“The checklist is easy to understand and accessible, but I wish it were detailed. The consideration of sustainable design expressions is controversial. For example, in the case of plant leather, natural materials are raw materials, but in some cases, the surface is plasticized to resemble leather during processing. It is said to be an effort toward sustainable development, but I think it may be risky.” (Interviewee D)

Suppose the constant update of design expression methods and numerical values for objective verification are supplemented. In that case, the checklist can be a practical method for designers to innovate or change sustainably. Furthermore, it can provide designers with in-depth sustainable knowledge if additional data on items that require discussion are provided.

## Discussion

This study identified a sustainable design practice method to satisfy customer sensibility and individual taste that designers need in the early stage of the SFD process. The SFB design approach was categorized through a literature review. Through the SFB case study, specific design expression methods for each category of the SFB design approach considering customer sensibility and personal taste were derived. The contents derived from this process were made into a checklist, and the design approach of Korean SFBs was confirmed through an interview.

It has been about 10 years since global brands in the fashion industry started to develop sustainability initiatives for a circular economy. As a result of case studies, 149 SFBs appeared in articles ranking the SFBs of the world’s top popular fashion magazines. In total, 35 brands emerged in the articles in 2019, 19 brands in 2020, and 56 brands in 2021. Although there was a market contraction due to Corona 19, the number of fashion brands increased significantly in 2021 is considered to be related to increased consumer interest in SFD. Given the weight of the impact of the fashion industry on the environment and the design method of a fashion designer can have an influence of 80% on the environment (Ribeiro et al., 2013; Ahmad et al., 2018), a specific SFD method considering the circular economy of products selected by consumers is required. In the sustainable fashion sector, the environmental impact is divided into the manufacturing phase of textile and apparel production and the transportation, product use, and end-of-life phases. In the end, the environmental impact depends on the lifespan of the product and the behavior of consumers, and it can be said that it is essentially caused by the production process in which the product is manufactured and the stage of use (Benkirane et al., 2022). From this point of view, this study focused on the sustainable design method of fashion products preferred by consumers. In other words, a design approach that meets the sensibility and taste of consumers is also related to product life extension, remanufacturing, and recycling, thereby forming a virtuous cycle structure of a circular economy.

In this study, in order to find a sustainable design method that consumers can like, a case study of specific design methods of SFBs appearing in the world’s top fashion magazines with many subscribers was conducted. Here, it was confirmed that various design approaches are used for each category proposed in previous studies as a design method for a sustainable circular economy.

In the “eco-friendly materials” design approach to maintain a sustainable raw material supply, “using certified sustainable materials,” “highly traceable and non-toxic material,” “dyeing process of green peace-certified,” “local or nearby materials,” and “using packages of organic materials” was applied in a specific way. “Functional durability design” that can reduce consumption, which is the ultimate goal of achieving a circular economy, was oriented toward slow manufacturing by “the quality and durability,” “on-demand production,” and “changeable design.” “Fashion for reuse and remanufacturing,” which aims to realize a sustainable circular economy through a virtuous cycle of resources, is the most well-known SFD approach. “Use recycled materials,” “sourcing 100% of synthetic fibers waste,” “upcycled waste clothing,” “repurposing,” and “repair and restoration” emerged as specific methods. A specific method that was impressive in the case study was “recycling of plastic bottles into yarn and fabric.” Recycling plastic bottles are being recycled in terms of circular economy theory and practice (Qu et al., 2019). Nevertheless, there are still negative views. In the report “Synthetics Anonymous (Changing Markets Foundation, 2021)” published by the Changing Markets Foundation (2021), downcycling plastic made from recycled plastic bottles, that is, clothes using recycled polyester, will eventually end up in landfill or incineration instead of circulating fashion. However, from the perspective of the circular economy, it is considered necessary to recycle the waste. Alternatives should be provided in the sense that today’s consumers’ product selection is determined by their sensibility and taste. Emotionally durable design is a design strategy that makes it possible to extend the life of a product by “strengthening the user-product relationship” (Norman, 2007; Chapman, 2009; Cooper, 2016). In particular, emotionally durable design has been proposed as an important principle of circular design by some scholars, but the concrete details of how emotional attachment and trust can be achieved in practice are not sufficiently presented (Haines-Gadd et al., 2018).

In the case analysis of this study, “emotionally durable design” appeared as “collaboration with local artisans,” “folklore handcrafted touches,” “handwoven material sourcing,” “personal design concept,” and “brand signature pieces.”

In an interview survey of SFBs in Korea, the approach of “emotionally durable design” was applied by only a few brands as a sustainable design method. Some of the participants even understood that this design approach was a sustainable design approach, after checking the checklist for a specific design approach of “emotionally durable design.” Compared to other design approaches, “emotionally durable design” is composed of abstract keywords, so it is considered that it is not well recognized according to individual characteristics.

Sustainable fashion technology, which reduces fabric loss, “seamless garment technology,” “digital printing to reduce water use,” and “combined laser robotics” appeared as SFD-specific approaches relatively few compared to other design approaches. The checklist of this study is meaningful in that it can be a tool for designers to easily reach the SFD approach in design practice. However, it is a limitation of the study that we were unable to include a large number of interviewees by conducting interviews with SFBs supported by the Korean government.

## Conclusion

This study aimed to identify a sustainable design practice. Based on an empirical case study with a theoretical background, a checklist was developed as a tool for sustainable fashion design methods. The inventory for the sustainable design approach suggested as a result of the case study is expected to provide an efficient design method by lowering barriers to practitioners who have had difficulty accessing the concept and design method of sustainable design.

In the sustainable design approach, some items need discussion according to the producer’s values. Concerns have been raised about the sustainability of fashion brands as a marketing tool in this regard. Accordingly, designers’ acquisition of sustainable knowledge is essential. Furthermore, it improves the emotional durability of fashion products, reflecting customer emotions and personal tastes, thereby increasing the sustainability of fashion products. Therefore, the designer’s active role is required. This study is significant in that it presents a checklist, an easy and efficient tool to address designers’ inadequate knowledge and lack of awareness of sustainability, and a sustainable design practice method that can satisfy customer emotions and personal tastes and improve environmental performance.

## Data availability statement

The original contributions presented in this study are included in the article/supplementary material, further inquiries can be directed to the corresponding author.

## Ethics statement

Ethical review and approval was not required for the study on human participants in accordance with the local legislation and institutional requirements. Written informed consent from the patients/participants or patients/participants legal guardian/next of kin was not required to participate in this study in accordance with the national legislation and the institutional requirements.

## Author contributions

YY contributed to the conception and design of the study. SK performed the interview and wrote sections of the manuscript. Both authors contributed to manuscript revision, read, and approved the submitted version.
